# Management of retinitis pigmentosa by Wharton’s jelly-derived mesenchymal stem cells: prospective analysis of 1-year results

**DOI:** 10.1186/s13287-020-01870-w

**Published:** 2020-08-12

**Authors:** Emin Özmert, Umut Arslan

**Affiliations:** 1 Faculty of Medicine Department of Ophthalmology, Ankara University, Ankara, Turkey; 2 Bioretina Eye Clinic, Ankara University Technopolis, Neorama Ofis 55-56 Yaşam Cad. No 13/A Beştepe, Yenimahalle, Ankara, Turkey

**Keywords:** Retinitis pigmentosa, Stem cell, Wharton jelly, Genotype, Autosomal dominant, Autosomal recessive

## Abstract

**Purpose:**

The aim of the study was to investigate annual structural and functional results, and their correlation with inheritance pattern of retinitis pigmentosa (RP) patients who were treated with Wharton’s jelly-derived mesenchymal stem cells (WJ-MSCs).

**Material and methods:**

This prospective, sequential, open-label phase-3 clinical study was conducted at Ankara University Faculty of Medicine, Department of Ophthalmology, between April 2019 and May 2020. The study included 34 eyes from 32 retinitis pigmentosa patients of various genotypes who were enrolled in the stem cells clinical trial. The patients were followed for 12 months after the WJ-MSCs transplantation into subtenon space and evaluated with consecutive examinations. Genetic mutations were investigated using a retinitis pigmentosa panel sequencing method consisting of 90 genes. All patients underwent a complete routine ophthalmic examination with best corrected visual acuity, optical coherence tomography angiography, visual field, and full-field electroretinography. Quantitative data obtained from baseline (T0), 6th month (T1), and 12th month (T2) examinations were compared.

**Results:**

According to timepoints at T0, T1, and T2: The mean outer retinal thickness was 100.3 μm, 119.1 μm, and 118.0 μm, respectively (*p* = 0.01; T0 < T1, T2). The mean horizontal ellipsoid zone width were 2.65 mm, 2.70 mm, and 2.69 mm respectively (*p* = 0.01; T0 < T1, T2). The mean best corrected visual acuity (BCVA) were 70.5 letters, 80.6 letters, and 79.9 letters, respectively (*p* = 0.01; T0 < T1, T2). The mean fundus perimetry deviation index (FPDI) was 8.0%, 11.4%, and 11.6%, respectively (*p* = 0.01; T0 < T1, T2). The mean full-field flicker ERG parameters at T0, T1, and T2: amplitudes were 2.4 mV, 5.0 mV, and 4.6 mV, respectively (*p* = 0.01; T0 < T1, T2). Implicit time were 43.3 ms, 37.9 ms, and 38.6 ms, respectively (*p* = 0.01; T0 > T1, T2). According to inheritance pattern, BCVA, FPDI, ERG amplitude, and implicit time data improved significantly in autosomal dominant (AD) and in autosomal recessive (AR) RP at 1 year follow-up (pAD = 0.01, pAR = 0.01; pAD = pAR > pX-linked). No ocular or systemic adverse events related to the surgical methods and/or WJ-MSCs were observed during the 1 year follow-up period.

**Conclusion:**

Subtenon transplantation of WJ-MSCs was found to be effective and safe in the treatment of RP during the first year, similar to the sixth month’s results. In autosomal dominant and autosomal recessive inheritance of RP, regardless of the genetic mutations, subtenon administration of WJ-MSCs can be considered an effective and safe option without any adverse effect for slowing or stopping the disease progression.

**Trial registration:**

ClinicalTrials.gov, NCT04224207. Registered 8 January 2020

## Introduction

Retinitis pigmentosa (RP) is a neurodegenerative genetic disorder characterized by progressive outer retinal degeneration with photoreceptor (PR) loss [1–4]. Light is absorbed by the retinal pigment epithelium (RPE), and the visual cycle occurs in the outer segments of the photoreceptors. In the visual cycle, rhodopsin–opsin and vitamin A are metabolized and regenerated. Rhodopsin–opsin proteins are genetically encoded in the inner segment of the photoreceptors. These proteins are wrapped in a disc-shaped lipoprotein membrane. Discs are sent to the outer segment of photoreceptors through the microtubules and cilia. In the RPE, peptide growth factors (GFs) are genetically encoded. Peptide growth factors in the RPE are responsible for the oxidative phosphorylation cycle, phagocytosis of metabolic wastes, and the transfer of vitamins and coenzymes. Some peptides are needed for recycle of rhodopsin–opsin metabolism [5–8]. To date, 90 genes for necessary protein synthesis have been identified at each stage of the visual cycle. Today, 260 different mutations detected in these 90 genes have been shown to lead to RP [9, 10]. Retinitis pigmentosa is a rare and very heterogeneous disease [11–22]. For this reason, gene therapy has many unknowns and is not cost-effective [10, 23]. Stem cell-based paracrine trophic therapies can be effective for a large number of patients regardless of the genetic inheritance [24–45]. For this purpose, stem cell research has increased in recent years, e.g., our previous study with Wharton’s jelly-derived mesenchymal stem cells (WJ-MSCs) in the treatment of RP patients [46].

The aim of the study was to investigate the annual structural and functional results of WJ-MSC treatment modality for RP and their relation with inheritance patterns.

## Materials and methods

Ethics committee approval for the umbilical cord Wharton’s jelly-derived mesenchymal stem cell (WJ-MSC) study was obtained from the Ankara University Faculty of Medicine Clinical Research Ethics Committee (19-1293-18) and was also approved by the Review Board of the Cell, Organ, and Tissue Transplantation Department within the Turkish Ministry of Health (56733164/203 E.507). The study was performed in accordance with the tenets of the 2013 Declaration of Helsinki. Written informed consent was obtained from the patients prior to enrollment.

This prospective, sequential, open-label clinical phase-3 study was conducted at Ankara University Faculty of Medicine, Department of Ophthalmology, between April 2019 and May 2020. Thirty-four eyes of 32 RP patients were included in the study. The primary diagnosis was confirmed by a genetic mutation RP panel test in addition to clinical findings. All patients enrolled in this study underwent a complete routine ophthalmic examination, including the best corrected visual acuity (BCVA) measurement with the early treatment of diabetic retinopathy study (ETDRS) chart (Topcon CC 100 XP, Japan). The patients were further evaluated with optical coherence tomography angiography (OCTA) from RTVue XR (Avanti, Optovue, Fremont, CA, USA) to confirm the diagnosis and to analyze and measure the changes in the retinal layers that provided a typical co-registered en-face and cross-sectional multimodal imaging platform. Structural examination of photoreceptors was followed by ellipsoid zone width (EZW) and outer retinal thickness (ORT) by the OCTA. Functional evaluation of photoreceptors was followed by Compass 24/2 visual field (VF) test (Compass, CenterVue, Padova, Italy) and full-field flicker electroretinography (ERG) device (RETeval, LKC Tech. Inc., Gaithersburg, MD, USA).

### Subjects

Thirty-four eyes of 32 RP patients were included in the phase-3 stem cell study.

Inclusion criteria consisted of several parameters:

- RP patients of any genotype and phenotype
- Patients with BCVA better than 50 letters
- Any degree of visual field loss
- Patients over 18 years old

Exclusion criteria consisted of several parameters:

- The presence of glaucoma and/or significant lens or vitreous opacities that may affect the measurement results of OCTA
- Presence of epilepsy-like systemic neurological disease that may affect the ERG results
- Pregnancy during study
- Smoking and/or receiving various vitamin supplements for RP

### Umbilical cord Wharton’s jelly-derived mesenchymal stem cells preparation

**Fig. 1:**
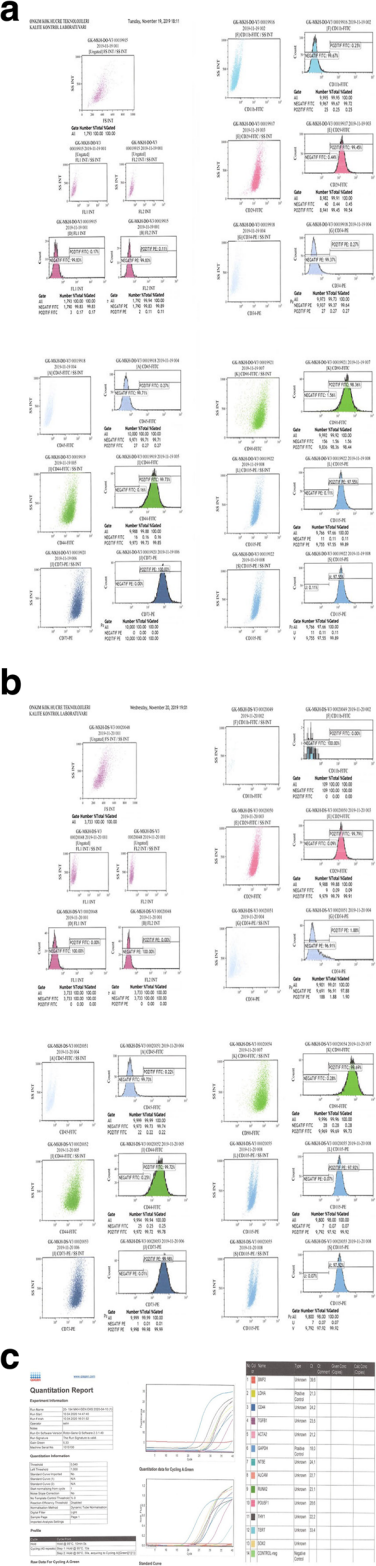
**a** The phenotypic characterization of Wharton jelly-derived mesenchymal stem cells before cryopreservation. **b** The phenotypic characterization of Wharton jelly-derived mesenchymal stem cells after cryopreservation. **c** The quantitative results of gene expression products of Wharton jelly-derived mesenchymal stem cells

The mesenchymal stem cells used in this study were isolated from Wharton’s jelly of the umbilical cord that was collected allogenicly from a single donor with the mother’s consent. The umbilical cord sample was treated following several steps. Briefly, cord tissue was washed twice with phosphate-buffered saline (Lonza, Switzerland) and the Wharton’s jelly part was minced using forceps and a scalpel. Minced pieces were cultivated in a cell culture dish (Greiner Bio-One, Germany) with Dulbecco’s modified Eagle’s medium F12 (DMEM)-low glucose with no L-glutamine (Biological Industries, Israel) and 10% human AB serum (Capricorn, Germany), 1% 10.000 U/mL penicillin, and 10.000 μg/mL streptomycin (Gibco, USA). All cell preparations and cultivation procedures were conducted in a current good manufacturing practice (cGMP)-accredited laboratory (Onkim Stem Cell Technologies, Turkey). The culture-expanded cells were cryopreserved at P3 using standard cryopreservation protocols until used in the following experiment. CryoSure-DEX40 (WAK-Chemie Medical, Germany) containing 55% dimethyl sulfoxide and 5% dextran 40 was used as cryopreservant. The cells were characterized at the time of cryopreservation using flow cytometric analysis to determine the expression of the positive cluster of differentiation (CD) surface markers, CD90, CD105, CD73, CD44, CD29, and negative for CD34, CD45, and CD11b (Fig. 1a, b). Using real-time polymerase chain reaction (qPCR), the expressions of several genes, such as tumor necrosis alpha (*TNF alpha*) and vimentin (*VIM*) were analyzed (Fig. 1c). Additionally, quality control analyses, such as mycoplasma and endotoxin analyses (using the PCR and LAL test combined with sterility analysis, respectively) were also completed. Cells were solubilized from cryopreservation before being prepared for injection. Average cell viability for each treatment was over 90.0%, and each patient received 2–6 × 10^6^ cells in a 1.5-ml saline solution.

### Injection of umbilical cord WJ-MSCs

The WJ-MSCs suspension from the culture was delivered to the operating room by cold chain and used within 24 h. A total of 1.5 ml of the WJ-MSC suspension was immediately injected into the subtenon space of each eye. The procedure was conducted under topical anesthesia with proparacaine hydrochloride drops (Alcaine, Alcon, USA) and sterile conditions. 5/0 atraumatic traction suture was applied to the limbus for easy access and manipulation to application area. Postoperatively, loteprednol, and tobramycin combination eye drops were given four times per day for 1 week, and oral amoxicillin clavulonate (1 g) was given twice a day for 5 days.

The patients were followed for 12 months after WJ-MSCs injections. The quantitative results were obtained by comparing the pre-injection and 6th and 12th month values. The main purpose of this clinical study was to evaluate the effects of WJ-MSCs on retinal structures and functions. The secondary purpose was to establish a correlation between the inheritance patterns and their responses to the WJ-MSCs transplantation.

### Timeframe

The patients were evaluated at several study timepoints:

- T0 (baseline): immediately before the WJ-MSC injection
- T1: 6th month after injection
- T2: 12th month after injection

### Primary outcome measure

- Ellipsoid zone widths (EZW, mm): EZW shows healthy photoreceptors and was both measured horizontally and vertically (HEZW and VEZW, respectively) on cross-sectional structural optical coherence tomography (SD-OCT). A manual segmentation program was used for the measurement of EZW. The intersection of the ellipsoid zone and the outer limiting membrane is marked as reference points for EZW measurements. Full screen magnification was used to prevent measurement errors and results correlated with visual field parameters.

### Secondary outcome measures

- ETDRS visual acuity: The visual acuity scores obtained from the T0, T1, and T2 examinations were analyzed and compared using statistical tests to determine effectiveness.
- Visual field sensitivity: Fundus perimetry deviation index (FPDI, %) and mean deviation (MD); FPDI and MD records were examined in the 24/2 visual field of the computerized perimetry records. The FPDI offers data explaining how many of the 100 flashing points and what percentage of the visual field could be correctly seen by the patient. MD is the deviation value in the visual field compared to normal eyes. The FPDI value is more sensitive than the MD value for retinal diseases. For VF analysis, in order to avoid mistakes during the test, practice rounds were carried out three times before the WJ-MSC injection into each eye.
- Outer retinal thickness (ORT, μm): ORT is the thickness from the outer plexiform layer to the Bruch membrane in the 3 × 3 mm of foveal area. The measurement is done automatically by the OCTA device.
- Full-field flicker ERG: Flicker electroretinography is a noninvasive objective test that measures the electrical activity of the retina in response to a light stimulus. The 30-Hz flicker ERG reveals responses from the cone bipolar cells. Full-field flicker ERGs were recorded without mydriasis using the RETeval system. The measurements were taken according to the instructions provided with the instrument for both eyes. We used the 16 Tds protocol, which combines implicit time and amplitude, to create a numerical result.

### Genetic analysis

The diagnosis of RP was made clinically after complete ophthalmological examination together with OCTA. The patients’ clinical and detailed family histories were obtained. In terms of syndromic RP, symptoms, such as hearing loss, polydactyly, and mental retardation, were investigated. After obtaining clinical data, the patient was referred to the medical genetic clinic for analysis. Blood samples were taken from patients and family members. Genetic mutations and inheritance patterns were investigated using a DNA RP panel sequencing method consisting of 90 genes.

### Definition of safety outcome

Intraocular/intraorbital mass lesion, inflammation, fibrosis, proptosis, diplopia, afferent pupillary defect, corneal/lenticular haze, ocular allergic reactions, intravitreal and/or subretinal hemorrhages, retinal artery/vein occlusions, optic nerve changes, macular edema, vitreoretinal interface alterations, retinal tear(s) or retinal detachment (exudative, rhegmatogenous), and intraocular pressure change from baseline (≤ 5 mmHg) were considered to be serious adverse ocular events. OCTA multimodal imaging and B-scan orbital ultrasonography were also used to detect and confirm the presence of complications during each study period examination. Systemic allergic reactions and anaphylaxis were considered to be systemic side effects.

### Statistical methods

Descriptive statistics are presented with frequency, percentage, mean, and standard deviation values. Repeated-measures analysis of variance test (rANOVA) was used to analyze the differences in HEZW, VEZW, ORT, BCVA, FPDI, MD, fulfilled ERG amplitude, and implicit time scores according to the T0, T1, and T2 times. In addition, comparisons were made according to inheritance pattern groups. A Sidak binary comparison test was used for measurement difference between groups. In addition, correlation analysis was used to examine the relationships among the T0, T1, and T2 times. In the study, *p* values < 0.05 were considered statistically significant (*α* = 0.05). Analyses were done with SPSS 22.0 package program.

## Results

The study included 34 eyes from 32 RP patients of various genotypes who were enrolled in this phase-3 clinical stem cell clinical trial. Of the 32 patients, 18 were male, and 14 were female. Median age was 39.7 years (range 19–59 years). The cases were evaluated for quantitative parameters at baseline and at the 6th and 12th months.

### The mean outer retinal thickness

**Fig. 2:**
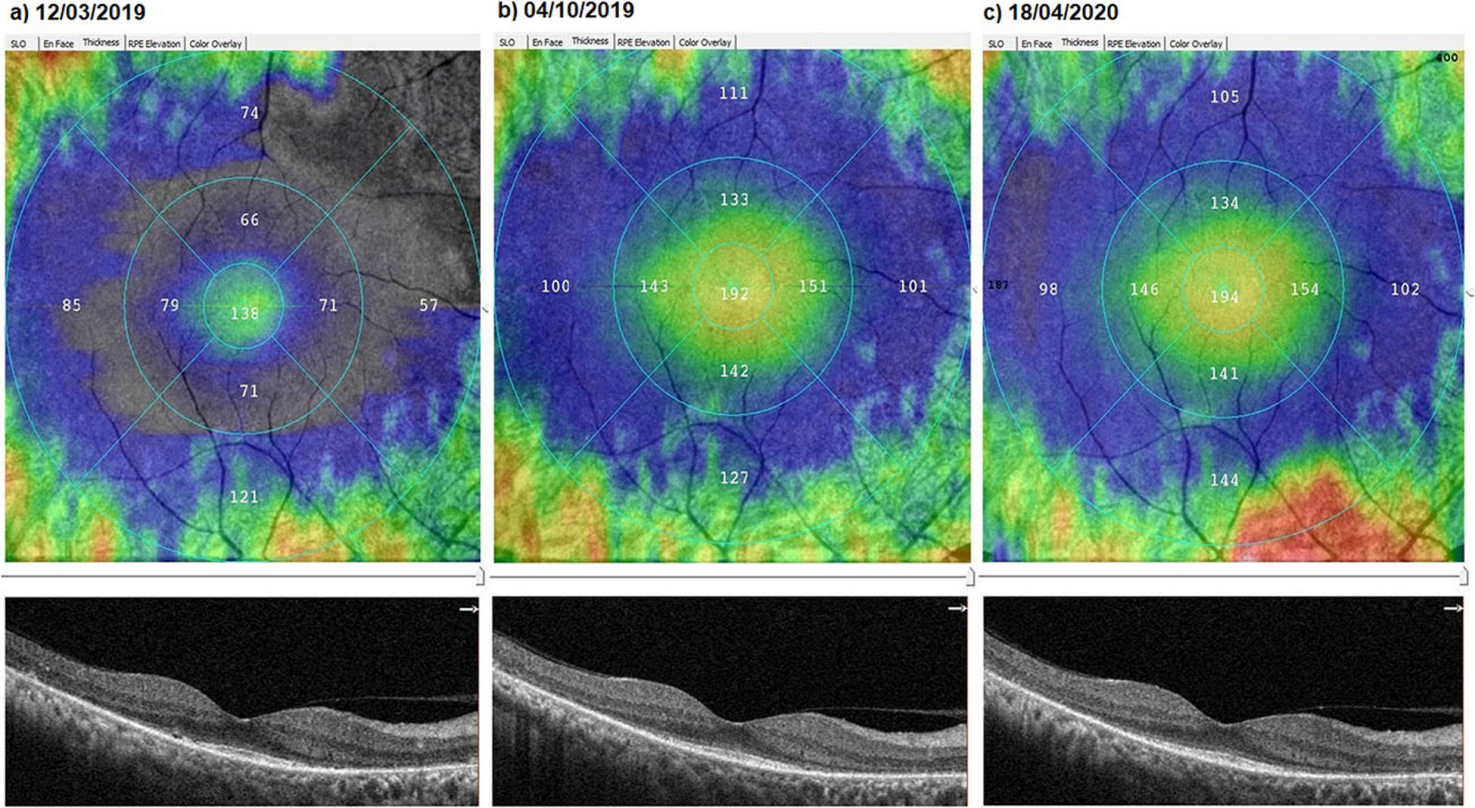
Improvement in “outer retinal thickness” according to study timepoints (T0, T1, T2) in the eye treated with the Wharton’s jelly-derived mesenchymal stem cell (WJ-MSC) (Table 1, patient 1: right eye—published data: **a** and **b** are the first 6-month changes of a patient from our previous study [46]). **a** Before application, 85.0 μm. **b** At 6th month, 152.2 μm. **c** At 12th month, 154.1 μm

**Fig. 3:**
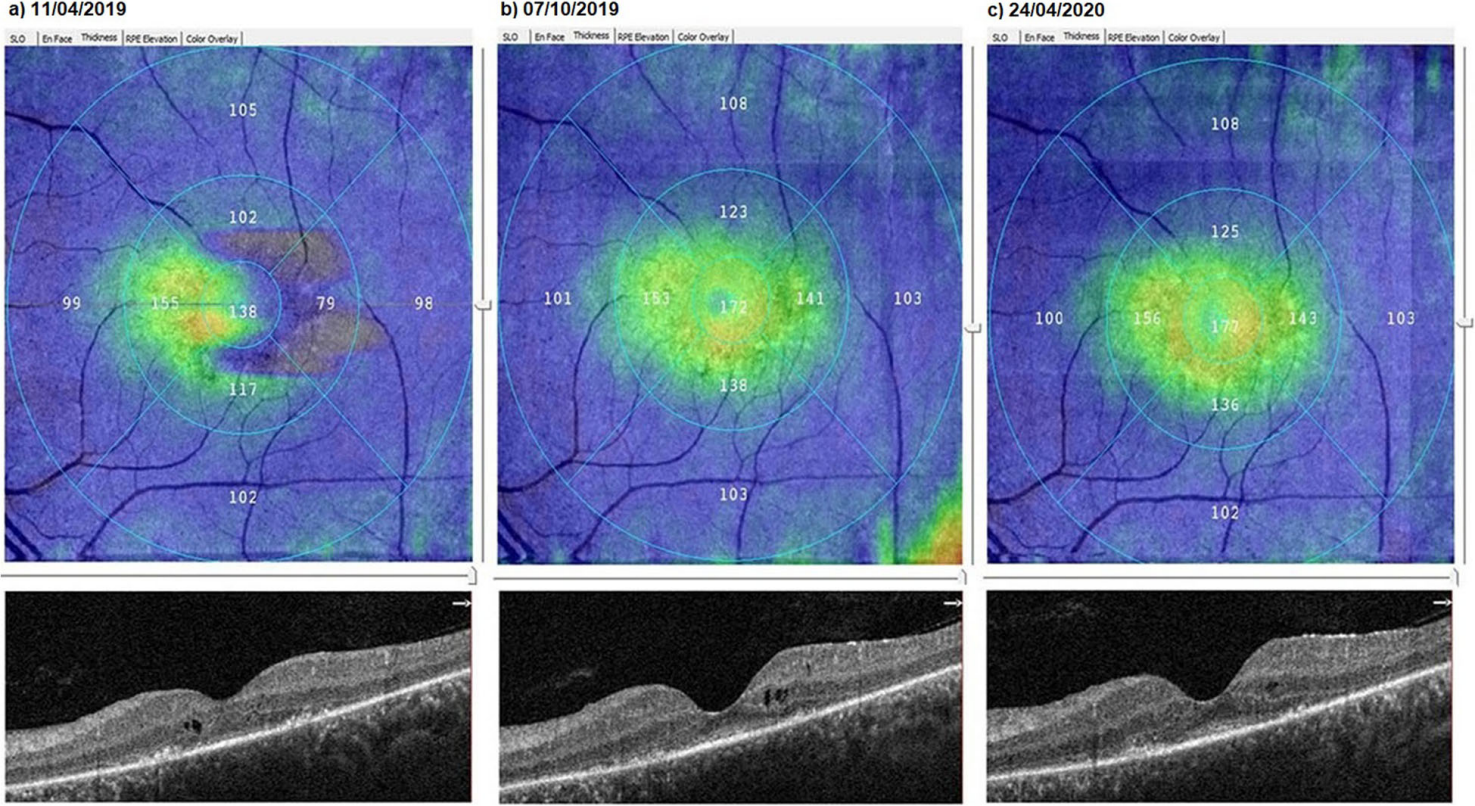
Improvement in “outer retinal thickness” according to study timepoints (T0, T1, T2) in the eye treated with WJ-MSCs (Table 1, patient 4: left eye—published data: **a** and **b** are the first 6-month changes of a patient from our previous study [46]). **a** Before application, 118.2 μm. **b** At 6th month, 145.4 μm. **c** At 12th month, 145.6 μm

**Fig. 4:**
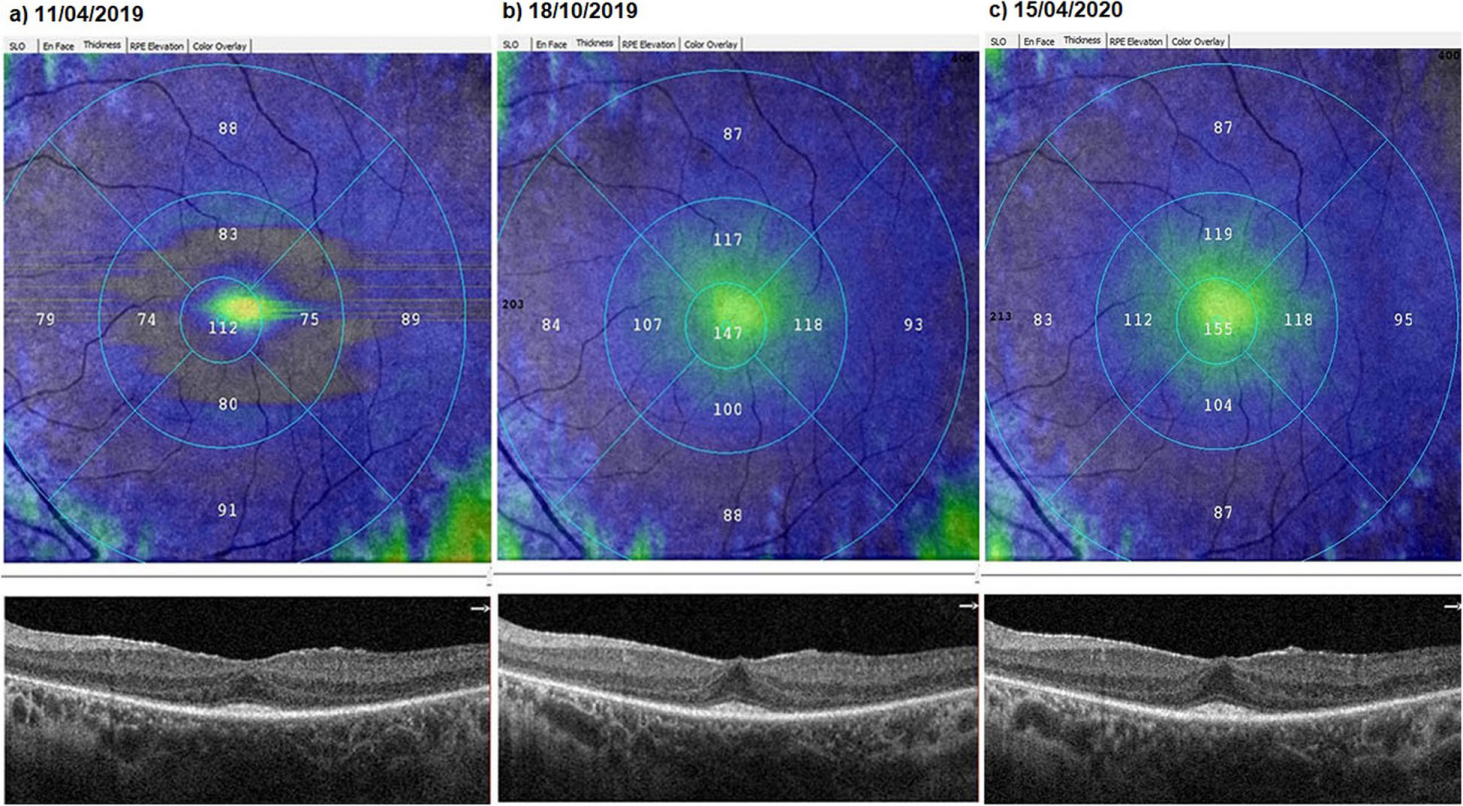
Improvement in “outer retinal thickness” according to study timepoints (T0, T1, T2) in the eye treated with WJ-MSCs (Table 1, patient 15: left eye). **a** Before application, 84.8 μm. **b** At 6th month, 120.0 μm. **c** At 12th month, 122.8 μm

At baseline, the ORT had a mean value of 100.3 μm before the WJ-MSCs application. At timepoint 1, the mean value was 119.1 μm after the 6-month application. At timepoint 2, the ORT had a mean value of 118.0 μm after the 12-month application (*p* = 0.01; T0 < T1, T2) as shown in Tables 1 and 3 and Figs. 2, 3, and 4.

**Table 1:** Change of the structural parameters of the retina according to study timepoints and genetic mutation analysis

Patient No	Genetic mutation	Eye	Horizontal EZW			Vertical EZW			Outer retinal thickness		
			T0	T1	T2	T0	T1	T2	T0	T1	T2
**1**	RHO	R	3.56	3.83	3.84	4.62	4.84	4.84	85.0	152.2	154.1
**2**	RP1	L	3.21	3.34	3.38	3.36	3.41	3.42	93.4	120.4	121.8
**3**	EYS	R	1.86	1.87	1.87	1.15	1.18	1.18	117.4	117.8	117.6
**4**	Unknown	L	3.93	4.14	4.12	2.72	2.74	2.74	118.2	145.4	145.6
**5**	RPGR	L	1.61	1.64	1.37	1.44	1.48	1.13	103.0	105.7	101.2
**6**	Unknown	R	2.74	2.75	2.77	2.49	2.51	2.51	129.0	131.9	132.4
**7**	C2ORF	R	4.01	4.09	4.10	4.02	4.08	4.11	116.0	126.3	126.6
**8**	USH2A	L	2.61	2.65	2.64	2.45	2.62	2.61	101.4	102.2	102.0
**9**	USH2A	L	3.91	3.98	3.98	3.92	3.96	3.96	79.2	135.6	134.4
**10**	RP1	L	3.90	3.99	3.98	3.86	3.92	3.91	105.0	111.8	110.9
**11**	PDE6B	R	3.87	3.87	3.86	3.71	3.70	3.69	92.2	128.7	127.8
**12**	USH2A	R	1.02	1.04	1.04	1.09	1.17	1.17	128.2	145.6	144.8
**13**	PDE6B	L	1.15	1.18	1.18	1.13	1.13	1.13	101.0	104.8	105.6
**14**	MERTK	R	2.23	2.25	2.25	2.08	2.12	2.12	100.4	110.0	110.1
**15**	PRPF3	L	2.18	2.22	2.23	2.14	2.20	2.21	84.8	120.0	122.8
**16**	RPGR	R	2.18	2.16	2.15	2.14	2.12	2.11	128.6	98.4	96.7
**17**	PDE6B	L	2.90	2.93	2.93	1.32	1.34	1.34	105.6	122.8	122.6
**18**	TULP1	L	1.42	1.48	1.48	1.25	1.25	1.25	89.0	94.8	96.0
**19**	USH2A	R	2.11	2.14	2.14	3.03	3.06	3.06	82.6	109.0	110.1
**20**	PDE6B	R	1.11	1.11	1.11	1.14	1.14	1.14	84.2	86.8	87.0
**21**	BBS2	R	1.15	1.18	1.18	1.06	1.08	1.08	85.2	97.0	97.3
**22**	PRPF3	L	2.49	2.49	2.49	2.26	2.26	2.26	86.0	116.0	115.7
**23**	RP1	R	2.51	2.51	2.51	2.54	2.54	2.54	91.0	91.6	91.0
**24**	RHO	L	3.18	3.24	3.24	3.21	3.34	3.35	118.2	152.4	152.6
**25**	RHO	L	3.26	3.28	3.28	3.14	3.16	3.16	106.0	132.8	132.7
**26**	PRPF3	L	**2.11**	**2.17**	**2.22**	2.29	2.34	2.34	109.3	122.0	122.1
**27**	Unknown	L	1.78	1.80	1.80	1.66	1.66	1.66	120.2	135.8	136.2
**28**	Unknown	R	1.66	1.65	1.65	1.38	1.37	1.37	116.0	122.8	120.7
**29**	EYS	R	1.48	1.48	1.47	1.51	1.50	1.50	117.0	117.0	116.8
**30**	RHO	L	4.71	4.98	5.02	4.79	4.86	4.91	110.0	140.4	141.7
**31**	RHO	R	4.90	4.90	4.90	3.96	3.96	3.96	101.0	106.0	106.3
	RHO	L	4.71	4.70	4.70	4.00	3.98	3.98	96.9	101.3	101.0
**32**	CERKL	R	2.57	2.60	2.60	2.44	2.44	2.44	70.4	111.8	111.6
	CERKL	L	1.98	2.00	1.98	1.87	1.87	1.86	72.6	112.4	112.0

*EZW* ellipsoid zone width (mm), *T0 (baseline)* just before the Wharton jelly-derived mesenchymal stem cell injection, *T1* 6th month after injection, *T2* 12th month after injection

### The mean horizontal ellipsoid zone width

**Fig. 5:**
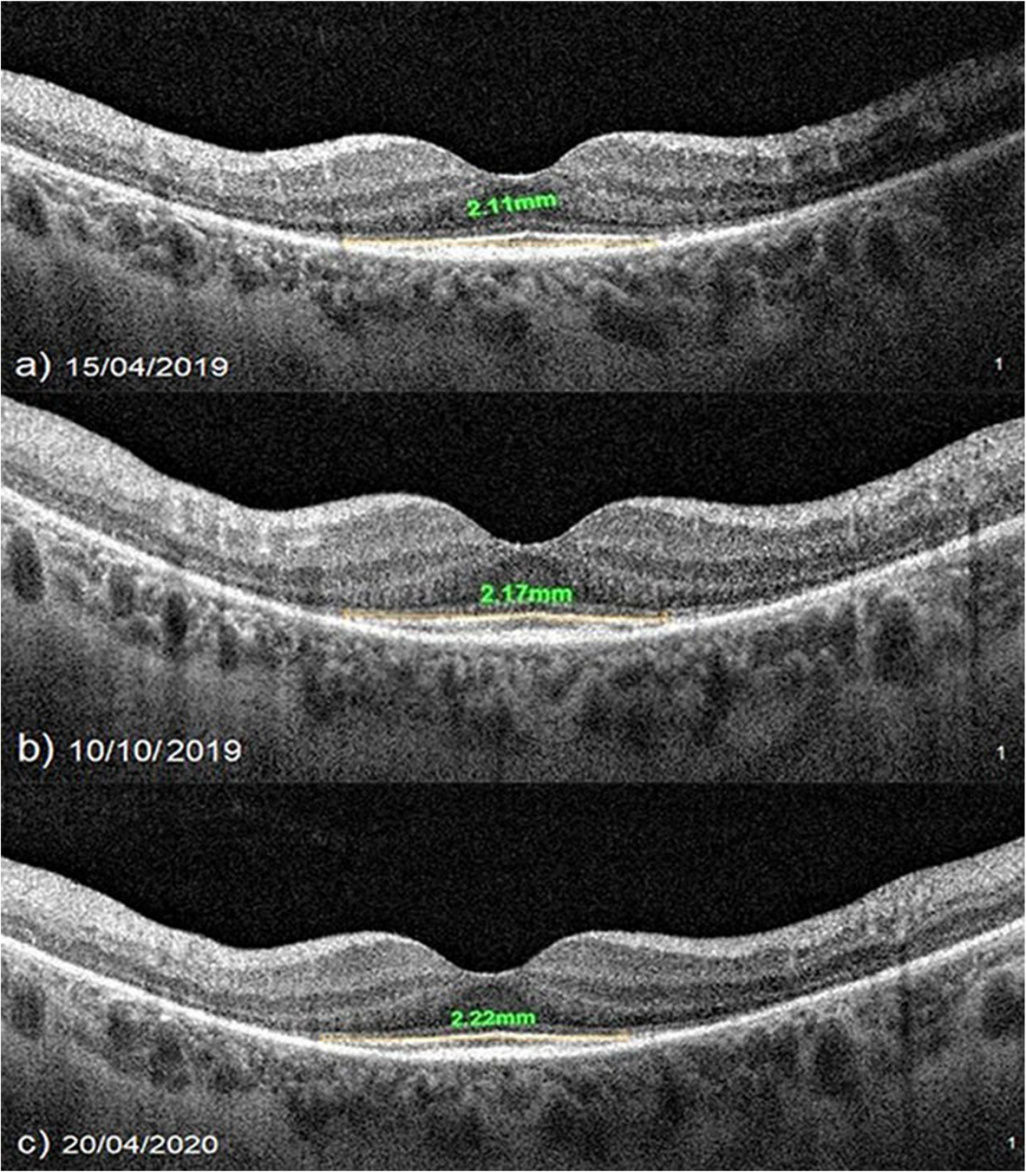
Increase in “horizontal ellipsoid zone width” according to study timepoints (T0, T1, T2) in the eye treated with WJ-MSCs (Table 1, patient 26: left eye). **a** Before application, 2.11 mm. **b** At 6th month, 2.17 mm. **c** At 12th month, 2.22 mm

At baseline, the HEZW had a mean value of 2.65 mm before the WJ-MSCs application. At timepoint 1, the mean value was 2.70 mm after the 6-month application. At timepoint 2, the HEZW had a mean value of 2.69 mm after the 12-month application (*p* = 0.01; T0 < T1, T2) as shown in Tables 1 and 3, and Fig. 5.

### The mean vertical ellipsoid zone width

**Fig. 6:**
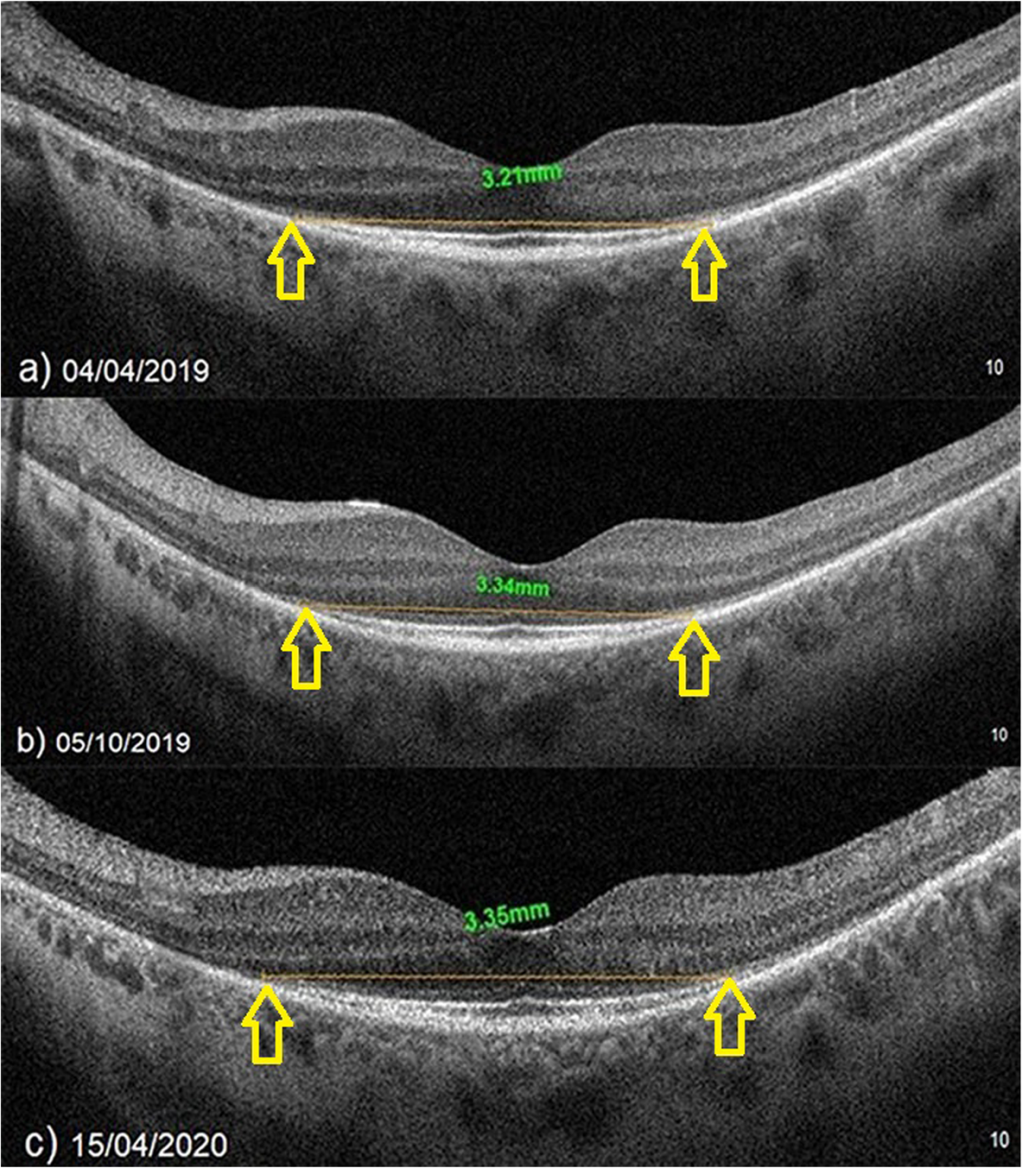
Increase in “vertical ellipsoid zone width” according to study timepoints (T0, T1, T2) in the eye treated with the WJ-MSCs (Table 1, patient 24: left eye—the points where the ellipsoid zone intersects the outer limiting membrane are marked as EZ length reference points, as indicated by the arrows). **a** Before application, 3.21 mm. **b** At 6th month, 3.34 mm. **c** At 12th month, 3.35 mm

**Fig. 7:**
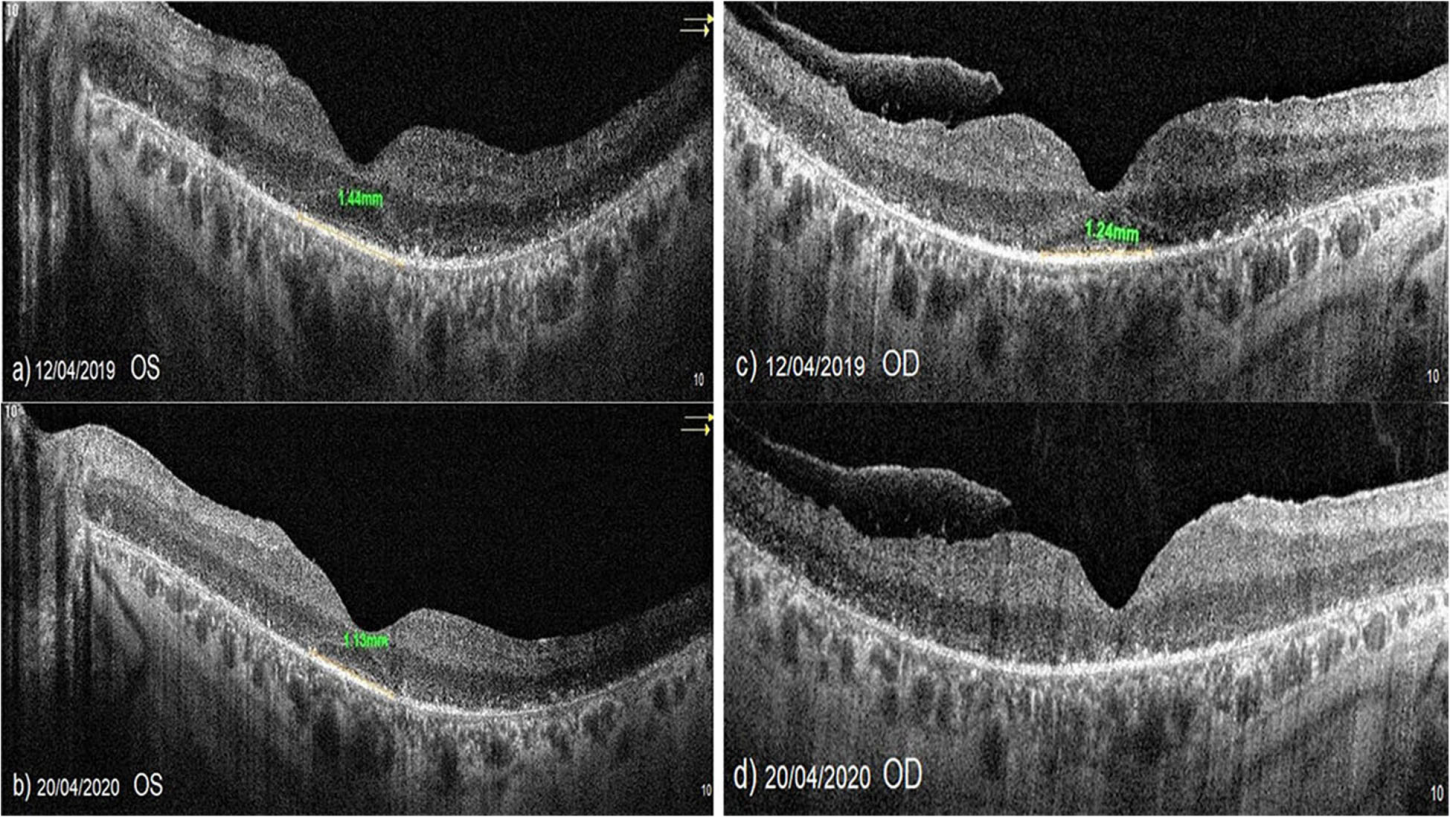
**a**, **b** Slowing in the “vertical ellipsoid zone width” loss according to study timepoints (T0, T1, T2) in the eye treated with the WJ-MSCs (Table 1, patient 5: left eye). **c**, **d** In the right eye of the same patient without WJ-MSCs treatment, “vertical ellipsoid zone width” decreased rapidly leading to blindness during the 1-year follow-up (X-linked retinitis pigmentosa, GTPase regulator [RPGR] mutation)

At baseline, the VEZW showed mean value of 2.51 mm before the WJ-MSCs application. At timepoint 1, was mean was 2.54 mm after the 6-month application. At timepoint 2, the VEZW had a mean value of 2.53 mm after the 12-month application (*p* = 0.08; T0 = T1 = T2) as shown in Tables 1 and 3, and Figs. 6 and 7.

### The mean best corrected visual acuity

At baseline, the BCVA had a mean value of 70.5 letters before the WJ-MSCs application. At timepoint 1, the BCVA had a mean value of 80.6 letters after the 6-month application. At timepoint 2, the BCVA had a mean value of 79.9 letters after the 12-month application (*p* = 0.01; T0 < T1, T2) as shown in Tables 2 and 3. In the autosomal dominant (AD) RP group, BCVA was mean 81.8 letters before WJ-MSC application and 91.6 letters after 1-year application (*p* = 0.01). In the autosomal recessive (AR) RP group, BCVA was mean 61.7 letters before application and 72.9 letters after 1-year application (*p* = 0.01). In the X-linked RP group, BCVA was mean 65.0 letters before application and 63.5 letters after 1 year application (*p* = 0.09) (pAR = pAD > pX-linked) as shown in Table 4.

**Table 2:** Change of the functional parameters of the retina according to study timepoints

Patient No.	Eye	BCVA			FPDI			ERG amplitudes			ERG implicit times		
		T0	T1	T2	T0	T1	T2	T0	T1	T2	T0	T1	T2
1	R	100	110	110	5	11	12	3.2	6.3	6.4	43.8	38.8	38.7
2	L	80	87	90	6	16	19	2.1	2.3	2.6	42.7	42.7	40.8
3	R	80	88	88	10	12	12	1.5	3.0	2.9	42.3	40.7	40.4
4	L	84	88	91	6	10	11	1.9	4.1	4.0	40.3	40.0	39.9
5	L	50	74	50	4	7	5	2.4	11.1	2.6	47.4	42.5	46.3
6	L	80	85	85	7	13	14	3.6	19.7	19.9	50.0	50.0	49.8
7	R	50	56	60	15	22	23	1.6	2.7	2.8	50.0	50.0	49.8
8	L	80	87	87	5	7	7	1.4	2.4	2.3	50.0	46.1	45.8
9	L	72	79	79	8	14	14	2.0	2.5	2.6	44.6	42.1	42.0
10	L	50	55	55	14	19	18	4.4	6.0	5.8	38.5	34.9	35.0
11	R	65	77	77	5	10	9	1.0	4.1	3.8	44.1	35.7	36.0
12	R	65	74	74	4	8	8	11.0	16.9	16.3	26.9	26.7	26.8
13	L	65	87	87	5	9	10	1.4	7.4	7.6	43.4	22.8	22.4
14	R	50	69	70	14	26	26	2.8	3.9	4.0	38.6	34.0	39.9
15	L	89	110	110	4	16	16	2.0	2.7	2.8	38.1	38.0	37.8
16	R	80	83	77	5	5	4	0.9	1.3	0.9	49.1	34.1	42.9
17	L	50	59	60	6	6	7	1.7	4.0	4.0	32.8	23.5	24.0
18	L	80	85	85	10	12	12	1.5	8.9	8.8	34.8	22.5	23.5
19	R	50	65	65	14	15	15	1.3	4.9	4.9	40.8	32.1	32.7
20	R	60	77	77	10	12	13	1.6	1.8	2.0	49.2	49.2	49.0
21	R	50	59	60	4	5	5	1.5	1.8	1.8	44.0	44.0	43.8
22	L	77	87	87	8	10	11	2.6	3.0	3.0	46.0	43.5	43.8
23	R	70	70	70	10	10	10	5.0	5.1	5.0	48.2	48.2	48.0
24	L	98	110	110	7	10	11	1.5	2.7	2.7	39.6	24.4	24.2
25	L	85	91	91	10	16	18	1.4	1.9	2.1	35.7	29.0	29.0
26	L	89	110	110	7	10	10	5.0	8.9	8.7	41.6	35.2	35.2
27	R	85	94	94	10	12	12	2.0	2.8	2.9	48.2	46.8	46.0
28	R	50	55	50	4	4	4	1.8	9.6	3.6	46.0	34.0	39.0
29	R	50	50	50	5	5	5	2.8	6.6	4.8	39.5	39.5	39.3
30	L	89	94	94	6	8	8	0.7	2.9	2.8	48.8	36.6	37.0
31	R	74	85	85	15	16	16	1.6	3.3	3.2	48.2	44.1	44.0
	L	80	87	87	14	15	15	2.0	2.8	2.8	47.4	43.2	43.2
32	R	60	77	77	8	11	11	1.9	2.0	2.0	44.0	36.0	37.0
	L	60	77	77	7	10	10	1.5	1.7	1.7	47.0	38.0	39.0

*BCVA* best corrected visual acuity (ETDRS letters), *FPDI* fundus perimetry deviation index (%), *ERG amplitudes* full-field flicker electroretinography amplitudes (mV), *ERG implicit times* full-field flicker electroretinography implicit times (ms), *T0 (baseline)* just before the Wharton jelly-derived mesenchymal stem cell injection, *T1* 6th month after injection, *T2* 12th month after injection

**Table 3:** Comparison of measurements according to study timepoints

Measurements	T0	T1	T2	*p*	Comparison**
	X ± s.s.	X ± s.s.	X ± s.s.		
ORT (μm)	101.29 ± 16.20	118.51 ± 17.36	118 ± 17.59	0.01*	T0 < T1, T2
HEZW (mm)	2.65 ± 1.12	2.70 ± 1.15	2.69 ± 1.16	0.01*	T0 < T1, T2
VEZW (mm)	2.51 ± 1.12	2.54 ± 1.14	2.53 ± 1.15	0.08	T0 = T1 = T2
BCVA (ETDRS)	70.5 ± 15.71	80.62 ± 16.26	79.97 ± 17.05	0.01*	T0 < T1, T2
FPDI (%)	8.00 ± 3.57	11.38 ± 4.84	11.59 ± 5.02	0.01*	T0 < T1, T2
ERG amplitude (mV)	2.37 ± 1.85	5.03 ± 4.21	4.53 ± 3.95	0.01*	T0 < T1, T2
ERG implicit time (ms)	43.28 ± 5.50	37.90 ± 7.88	38.58 ± 7.73	0.01*	T0 > T1, T2

**Repeated-measures analysis of variance test (rANOVA); *p < 0.05, statistically significant

*HEZW* horizontal ellipsoid zone width (mm); *VEZW* vertical ellipsoid zone width (mm); *ORT* outer retinal thickness (μm); *BCVA* best corrected visual acuity (ETDRS letters); *FPDI* fundus perimetry deviation index; *ERG amplitude* full-field flicker electroretinography; amplitude (mV); *ERG implicit time* full-field flicker electroretinography, implicit time (ms); *T0 (baseline)* just before the Wharton jelly-derived mesenchymal stem cell injection; *T1* 6th month after injection; *T2* 12th month after injection

**Table 4:** Comparison of functional measurements according to inheritance pattern

Inheritance	BCVA			FPDI		
	T0	T2	***p***	T0	T2	***p***
**AD** ***n*** **= 12**	81.8 ± 11.8	91.6 ± 12.2	0.01*	8.9 ± 2.7	13.2 ± 2.9	0.01*
**AR** ***n*** **= 16**	61.7 ± 10.9	72.9 ± 10.5	0.01*	8.1 ± 2.7	11.7 ± 2.7	0.01*
**X-linked** ***n*** **= 2**	65.0 ± 0	63.5 ± 0	0.09	4.5 ± 0	4.5 ± 0	0.1
Comparison**	pAR = pAD > pX-linked			pAD > pAR > pX-linked		

**Sidak binary comparison test; *p < 0.05, statistically significant

*AD* autosomal dominant, *AR* autosomal recessive, *X-linked* X-related inheritance pattern, *BCVA* best corrected visual acuity (ETDRS letters), *FPDI* fundus perimetry deviation index (%), *T0 (baseline)* just before the Wharton jelly-derived mesenchymal stem cell injection, *T2* 12th month after injection

### The mean visual field sensitivity

**Fig. 8:**
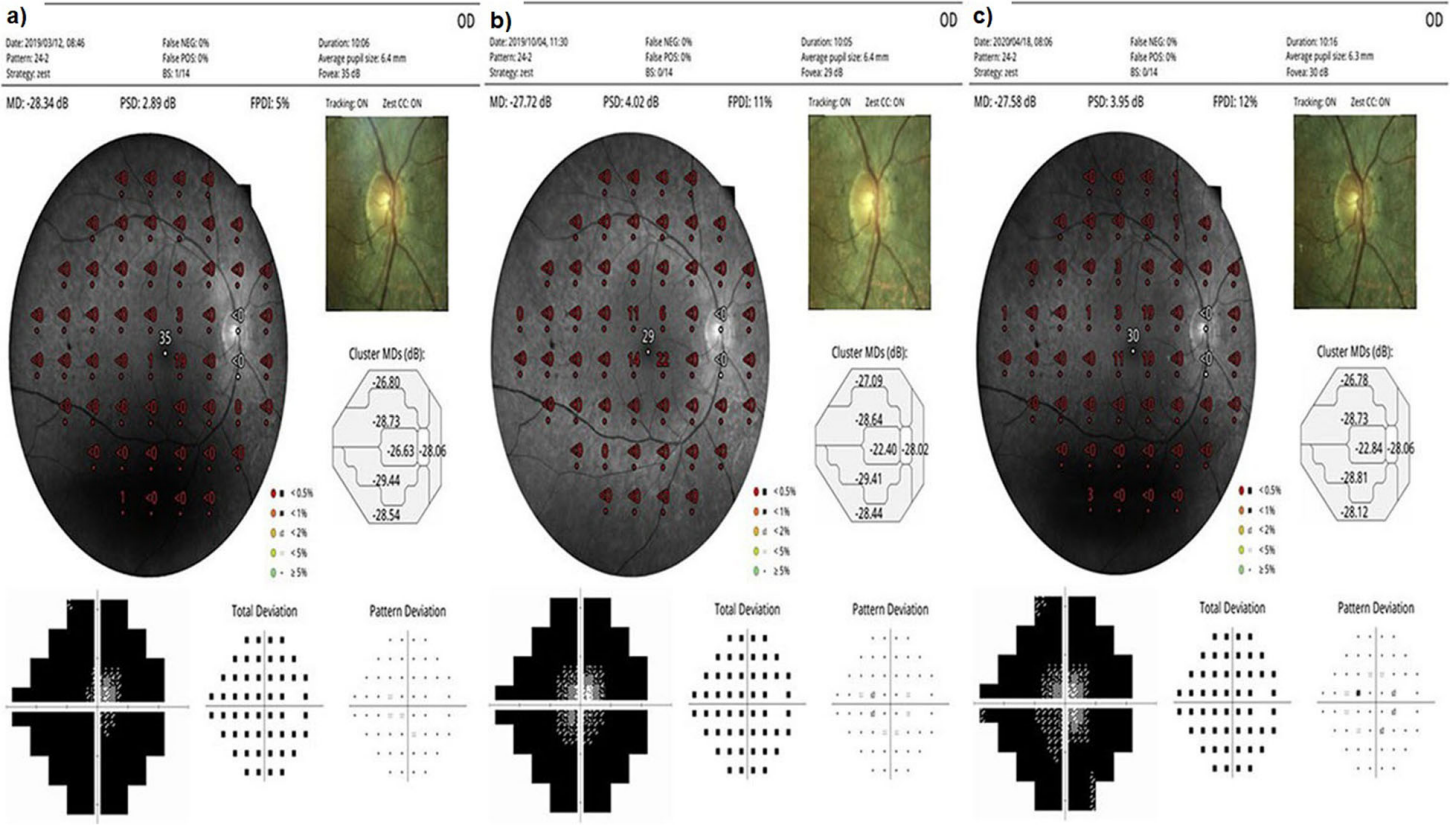
“Visual field” enlargement according to study timepoints (T0, T1, T2) in the eye treated with WJ-MSCs. Note the change in FPDI and MD values (Table 1, patient 1: right eye—published data: **a** and **b** are the first 6-month changes of a patient from our previous study [46]). **a** Before application, FPDI 5%—MD 28.34. **b** At 6th month, FPDI 11%—MD 27.72. **c** At 12th month, FPDI 12%—MD 27.58

**Fig. 9:**
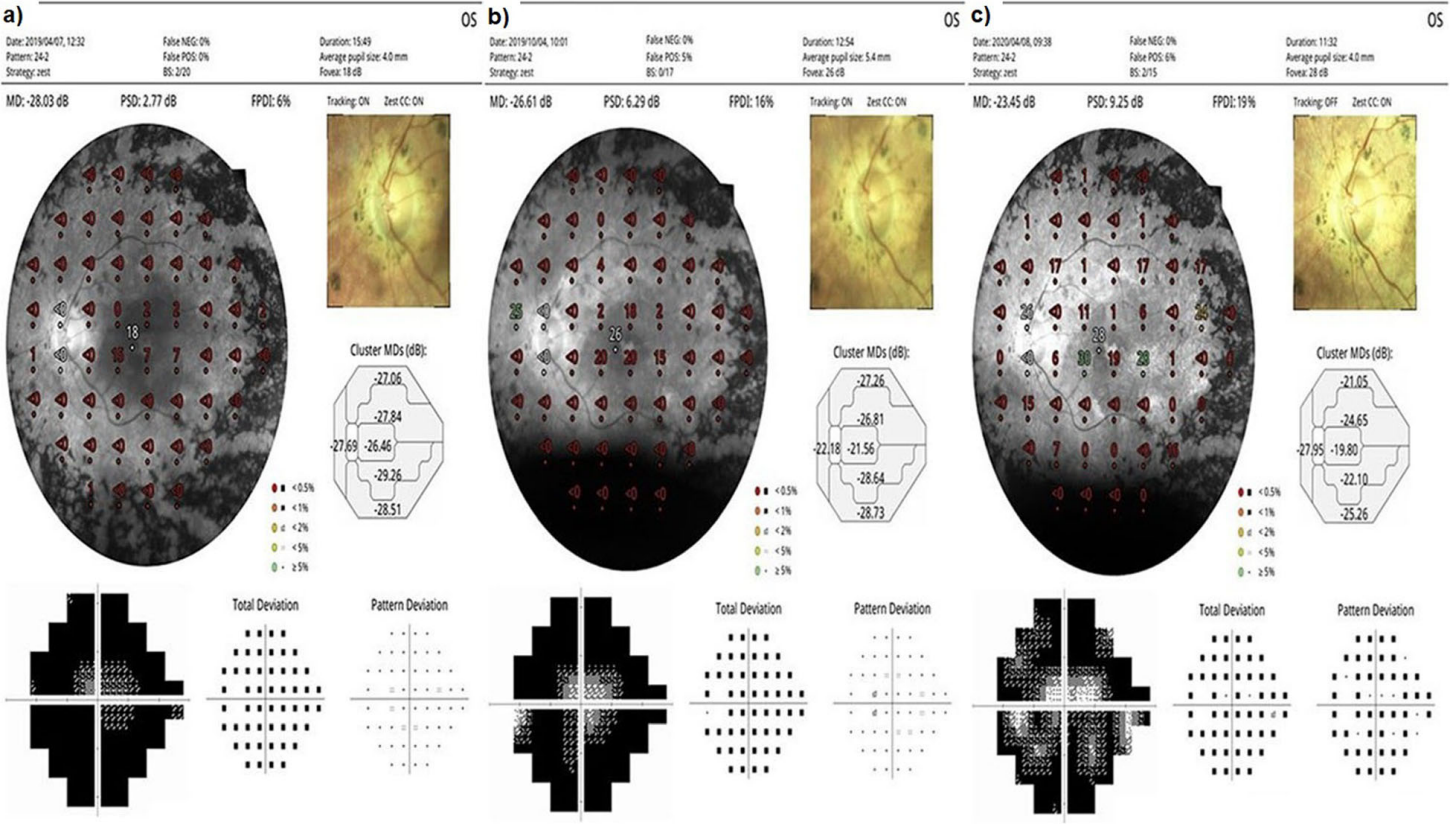
“Visual field” enlargement according to study timepoints (T0, T1, T2) in the eye treated with WJ-MSCs. Note the change in FPDI and MD values (Table 1, patient 2: left eye—published data: **a** and **b** are the first 6-month changes of a patient from our previous study [46]). **a** Before application, FPDI 6%—MD 28.03. **b** At 6th month, FPDI 16%—MD 26.61. **c** At 12th month, FPDI 19%—MD 23.45

**Fig. 10:**
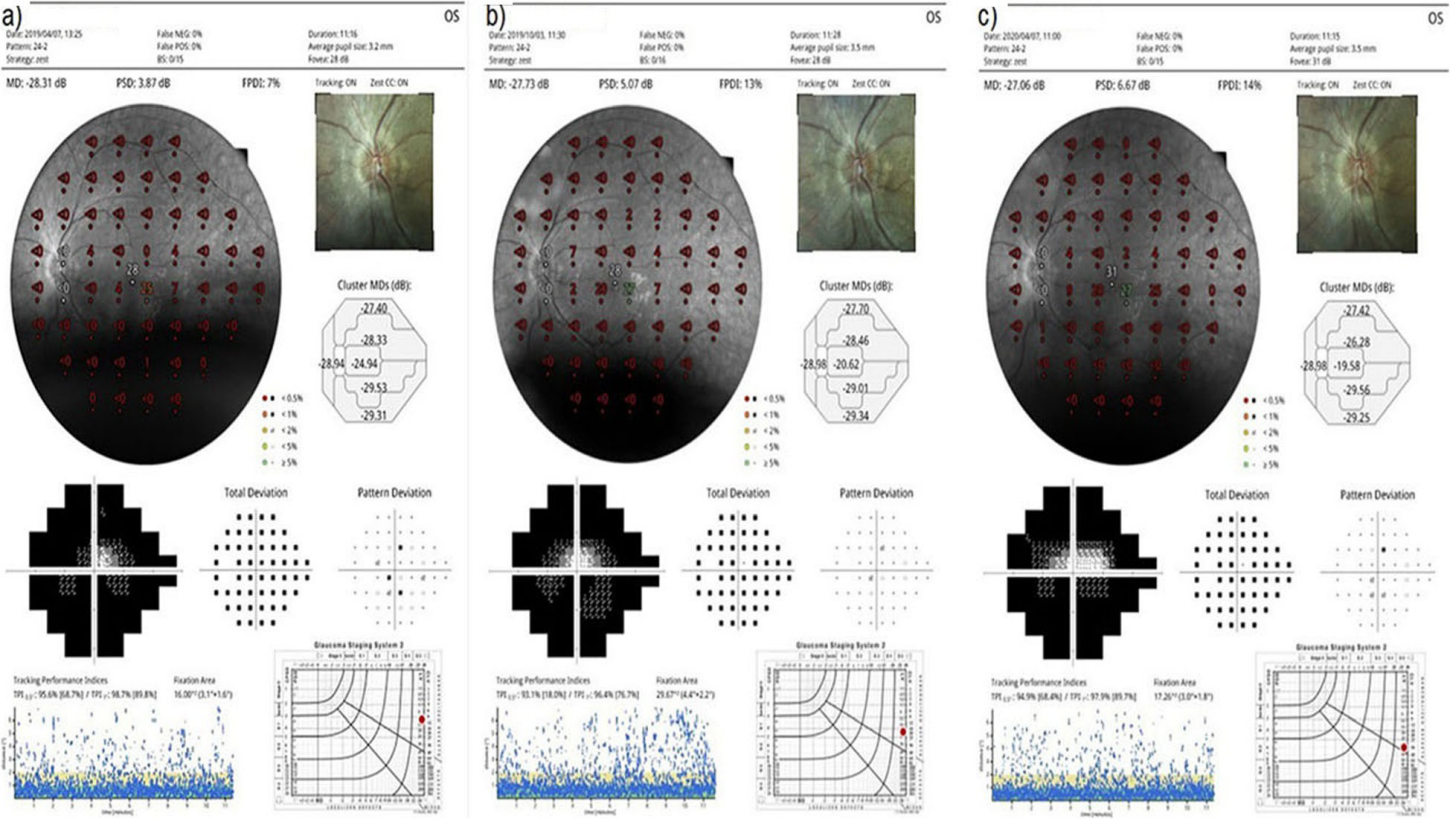
“Visual field” enlargement according to study timepoints (T0, T1, T2) in the eye treated with WJ-MSCs. Note the change in FPDI (Table 1, patient 6: left eye). **a** Before application, FPDI 7%. **b** At 6th month, FPDI 13%. **c** At 12th month, FPDI 14%

At baseline, the FPDI had a mean value of 8.0% before the WJ-MSCs application. At timepoint 1, a mean value of 11.4% after the 6-month application was seen. At timepoint 2, the FPDI had a mean value of 11.6% after the 12-month application (*p* = 0.01; T0 < T1, T2) as shown in Tables 2, 3, 4, and Figs. 8, 9, and 10. At baseline, the visual field mean deviation (MD) value was 27.3 dB before WJ-MSC application and 24.7 dB after the 6-month application and 24.6 dB after the 12-month application (*p* = 0.01; T0 < T1, T2). In the AD-RP group, FPDI was mean 8.9% before WJ-MSC application and 13.2% after 1-year application (*p* = 0.01). In the AR-RP group, FPDI was mean 8.1% before application and 11.7% after 1-year application (*p* = 0.01). In the X-linked RP group, FPDI was mean 4.5% before application and 4.5% after 1 year application (*p* = 0.1) (pAD > pAR > pX-linked) as shown in Table 4.

### The mean full-field flicker ERG parameters

At baseline, the amplitude presented a mean value of 2.4 mV before the WJ-MSC application. At timepoint 1, the mean ERG value was 5.0 mV after the 6-month application. At timepoint 2, the amplitude had a mean value of 4.6 mV after the 12-month application (*p* = 0.01; T0 < T1, T2). At baseline, the implicit time had a mean value of 43.3 ms before the WJ-MSC application. At timepoint 1, the mean value of this parameter was 37.9 ms after the 6-month application. At timepoint 2, the implicit time had a mean value of 38.6 ms after the 12-month application (*p* = 0.01; T0 > T1, T2), as shown in Tables 2 and 3. In the AD-RP group, ERG amplitude was mean 2.6 mV before WJ-MSC application and 3.9 mV after 1-year application (*p* = 0.01). In the AR-RP group, ERG amplitude was mean 2.3 mV before application and 4.5 mV after 1 year application (*p* = 0.01). In the X-linked RP group, ERG amplitude was mean 1.6 mV before application and 1.8 mV after 1 year application (*p* = 0.08) (pAR > pAD > pX-linked) as shown in Table 5. In the AD-RP group, ERG implicit time was mean 43.2 ms before WJ-MSC application and 38.1 ms after 1 year application (*p* = 0.01). In the AR-RP group, ERG implicit time was mean 42.0 ms before application and 36.9 ms after 1 year application (*p* = 0.01). In the X-linked RP group, ERG implicit time was mean 48.3 ms before application and 44.6 ms after 1 year application (*p* = 0.04) (pAR = pAD > pX-linked) as shown in Table 5.

**Table 5:** Comparison of functional measurements according to inheritance pattern

Inheritance	ERG amplitudes			ERG implicit times		
	T0	T2	***p***	T0	T2	***p***
**AD** ***n*** **= 12**	2.6 ± 1.6	3.9 ± 1.8	0.01*	43.2 ± 4.7	38.1 ± 4.8	0.01*
**AR** ***n*** **= 16**	2.3 ± 1.7	4.5 ± 1.7	0.01*	42.0 ± 4.4	36.9 ± 4.6	0.01*
**X-linked** ***n*** **= 2**	1.6 ± 0	1.8 ± 0	0.08	48.3 ± 0	44.6 ± 0	0.04*
Comparison**	pAR > pAD > pX-linked			pAR = pAD > pX-linked		

**Sidak binary comparison test; *p < 0.05, statistically significant

*AD* autosomal dominant; *AR* autosomal recessive; *X-linked* X-related inheritance pattern; *ERG amplitude* full-field flicker electroretinography, amplitude (mV); *ERG implicit time* full-field flicker electroretinography, implicit time (ms); *T0 (baseline)* just before the Wharton jelly-derived mesenchymal stem cell injection; *T2* 12th month after injection

### Genotypes

Genetic mutations could be detected in 28 of 32 patients as a result of RP panel sequencing. According to RP panel sequencing results, several gene mutations were found: (1) rhodopsin (*RHO*) in five patients, (2) pre-mRNA processing factor 3 (*PRPF3*) in three patients, (3) retinitis pigmentosa 1 (*RP1*) in three patients, (4) usherin 2A (*USH2A*) in four patients, (5) phosphodiesterase 6B (*PDE6B*) in four patients, (6) eyes shut homolog (*EYS*) in two patients, (7) tyrosine protein kinase Mer precursor (*MERTK*) in one patient, (8) ceramide kinase-like (*CERKL*) in one patient, (9) tubby protein homolog 1 (*TULP1*) in one patient, (10) Bardet-Biedle syndrome 2 (*BBS2*) in one patient, (11) C2ORF in 1 patient, and (12) retinitis pigmentosa GTPase regulator (RPGR) mutations in two patients were detected (Table 1). Whole exome sequencing was requested for four patients, in whom no mutation was detected by RP panel sequencing. Results for four patients have not been completed yet. Autosomal dominant (AD) inheritance pattern was detected in 39% of cases (RHO, PRPF3, RP1). Autosomal recessive (AR) inheritance pattern was detected in 54% of cases (USH2A, PDE6B, EYS, MERTK, CERKL, TULP1, BBS2, C2ORF). X-linked inheritance pattern was detected in 7% of cases (RPGR).

No serious ocular or systemic adverse events were encountered during the 1-year follow-up in any group related to WJ-MSCs applications. None of the patients had any mass lesions or fibrosis at the 1-year follow-up.

## Discussion

Genetic mutations that lead to retinitis pigmentosa can occur or be transferred by autosomal dominant (AD), autosomal recessive (AR), X-linked, mitochondrial, mosaic, or sporadic inheritance [9–22]. Rhodopsin and opsin are the major protein products which synthesized in the photoreceptors [18–20]. Photoreceptors also need lipoproteins and glycoproteins, which are part of the disc membranes for the transfer of functional proteins involved in the visual cycle [13–15]. The inner and outer segments of the photoreceptors are interconnected by cilia that have a microtubule structure. Cilia provide the transport of discs from the inner to the outer segment of photoreceptors. A high level of GTP–ATP is needed for this transport. The GTP transferase protein responsible for GTP formation is another major protein involved in the visual cycle [16, 17]. Structural and functional retinal proteins are synthesized in the RPE. The synthesis of growth factors responsible for oxidative phosphorylation and the energy cycle proteins, such as GDP reductase–GTP transferase, is the major visual cycle process in the RPE [5–8]. Peptides required for the removal of metabolic wastes by autophagy are also synthesized in RPE [47–50]. All of these structural and functional proteins are encoded by 90 genes that have been identified in the photoreceptors and RPE [51, 52].

WJ-MSCs possess a high paracrine trophic effect and secrete exosomes into the microenvironment (e.g., choriocapillaris–Bruch’s membrane–RPE and PR). These secreted exosomes include growth factors, cytokines, mRNA sequences, microRNA, and mitochondrial components [53–61]. The growth factors and mitochondrial content of exosomes stimulate cellular oxidative phosphorylation and the cellular energy cycle, thus, providing regenerative and restorative effects for the microenvironment [62, 63]. Neural growth factor (NGF), brain-derived neurotrophic factor (BDNF), and ciliary neurotrophic factor (CNTF) are the main exosome contents that affect the outer retinal layers. These growth factors also accelerate intracellular metabolism and autophagy [64–67]. Prostaglandin E2 (PGE2) and transforming growth factor β (TGF-β) in the exosome contents can prevent inflammation by regulating T and B lymphocyte functions [34]. Both acceleration of autophagy and immunomodulating cytokines can prevent inflammation-induced apoptosis. The mRNA and microRNA contents of the exosomes allow protein synthesis to be encoded by neighboring cells [55–58]. WJ-MSCs have some advantages over other stem cell preparations. WJ-MSCs do not cause ethical problems since it is obtained from medical waste of delivery such as placenta/umbilical cord. Cell surface antigens do not cause tissue rejection reactions and therefore do not require tissue compatibility [33–37]. They can be cultured in the relatively hypovascular subtenon cavity, which is quite safe compared to subretinal or intravitreal applications. Subtenon administration allows the growth factors secreted by WJ-MSCs to pass into the choroidal matrix through the scleral pores, and from there, they pass through the tyrosine kinase receptors to the subretinal space [68–71].

In autosomal dominant inherited RP (AD-RP), mutations in one of the two DNA alleles lead to the formation of dysfunctional mutant proteins. These proteins have a longer or shorter chain structure compared to normal proteins. The high level of mutant proteins with folding problems in the cell becomes difficult to remove by phagocytosis and autophagy [18–20]. If the level of mutant proteins in the cell reaches to toxic levels, oncosis (swelling), inflammation, and apoptosis mechanisms are triggered [72]. Since one of the two DNA alleles is intact in autosomal dominant (AD) mutations, cellular functions will proceed slowly. Photoreceptor loss rate is slow due to the presence of some natural proteins in the cell [23]. Annual average ellipsoid zone loss is reported as 5% in AD-RP [11, 12, 18–20]. Exosomes produced by stem cells induce autophagy, thus accelerating the intracellular clearance of mutant proteins. In this way, induction of oncosis, inflammation, and apoptosis mechanisms can be prevented [23]. In our study, we observed that the AD-RP group responds well to the WJ-MSC treatment in 1-year follow-up period.

In autosomal recessive inheritance RP (AR-RP), mutations are generally related to the energy cycle in RPE. Since both alleles are mutants, they progress faster. The average annual EZ loss rate is around 10% [11–17]. The proteins responsible for the energy cycle are similar to the exosome contents produced by stem cells [24–46]. Mutant proteins can be obtained from stem cells. In our study, the high electrophysiological response, especially in *PDE6B* and *CERKL* mutations, supports the idea that these effects may be related to the increase in cellular energy cycle. In our study, we observed that AR-RP group respond well to the WJ-MSC treatment in 1-year follow-up period.

In X-related RP, GTPase regulator mutations and cilia functions are degraded at an early age. Discs accumulate in the photoreceptor inner segment. Oncosis, inflammation, and apoptosis start at an early age. Annual loss of EZ is around 15% per year [11–15, 21, 22]. The GTPase regulator is a retina-specific protein, similar to RPE65 [73, 74]. We observed that two patients with X-linked RPGR mutation had a mild response to WJ-MSCs treatment during the first 6 months. The paracrine effects of WJ-MSCs can affect the general functions of neighboring cells. General functions of WJ-MSCs are the acceleration of the energy cycle, the acceleration of autophagy, the suppression of inflammation, and the prevention of apoptosis [47–52]. Retina-specific protein synthesis, such as the GTPase regulator, cannot be provided by stem cells. In our two patients with RPGR mutation, we observed that the response to stem cell therapy was not sufficient unlike other inheritance patterns. Gene therapy may be more effective than stem cells in retinal-specific protein mutations. However WJ-MSCs can be partially beneficial due to the effects of acceleration of autophagy and suppression of inflammation. In a case with X-linked RP, we observed a rapid loss of EZ in the untreated side eye (Table 1, Patient 5, Fig. 7). We observed that EZ loss was slower in the WJ-MSC-treated side eye. Although different genetic mutations may respond differently to treatment, WJ-MSCs appears to be an effective and safe treatment option without any complications that is compatible with RP pathogenesis.

Genetic mutations in RP lead to an impaired balance in the subretinal microenvironment. Photoreceptors and RPE solidify their cytoplasm for the delay the transition to apoptosis. The outer retinal layer cells cease all metabolic activities until the microenvironmental balance improves. Cells at this stage are defined as dormant phase; cells are alive but exist in sleep mode [75, 76]. At this stage, the EZ is shorter and the ORT is thinner than normal [77–79]. Structural measurements, such as EZW and ORT, and functional measurements such as BCVA, FPDI, and ERG responses can indicate photoreceptor reactivation (Tables 3, 4, and 5). Statistically significant improvements in these parameters show that the paracrine trophic effects of WJ-MSCs still persist during the first year similar to the results at 6th month [46]. Dormant phase reactivation and statistically significant increases were detected in 19 of 34 eyes (55.9%) according to visual field and EZW parameters. In 13 of 34 eyes (38.3%), the current condition could be preserved in the first year, and the disease remained stable. EZ loss was observed in the first year in two of 34 eyes (5.8%). Patients with loss in EZW were X-linked RPGR mutations, and the rate of loss was found to be slower in the treated eyes than in the non-treated side. In the first year, none of the patients had systemic or ocular serious adverse effects, such as orbital mass, rejection reaction, fibrosis, glaucoma, and/or uveitis.

Bone marrow-derived mesenchymal stem cells (BM-MSC) and adipose mesenchymal stem cells are other types of cells that have been tried in retinal dystrophies. BM-MSC caused intense inflammation and fibrosis in the retina [28, 29]. The duration of action of adipose cells was as short as 10 months [41–43]. For this reason, WJ-MSCs are evaluated as advantageous in many aspects compared to other cell types [33–36].

There are some limitations in the study. In RPE and photoreceptors, intracellular mutant protein deposits could be detected by fundus autofluorescence (FAF). Autophagy induced by the WJ-MSC may indicate changes in these deposits. Follow-up and evaluation with FAF may be a separate research topic. It is stated in preclinical studies that WJ-MSCs can proliferate with mitosis for about 4 years without any karyotypic changes [38, 39]. The question of how long the paracrine effects will last in the tissue and whether downregulation will develop in the tyrosine kinase receptors. These are separate research topics.

## Conclusion

In the treatment of RP, subtenon WJ-MSCs transplantation was found to be effective and safe in the first year, similar to the sixth month, presenting no adverse effects. In autosomal dominant and in autosomal recessive inheritance patterns, regardless of the genetic mutations, subtenon WJ-MSC transplantation can be considered an effective and safe option for slowing or stopping disease progression, based on this phase-3 clinical trial stem cell study results.

## Data Availability

The datasets generated during and/or analyzed during the study are available from the corresponding author on reasonable request.

## Abbreviations

BCVA: Best corrected visual acuity
BDNF: Brain-derived neurotrophic factor
cGMP: Current good manufacturing practice
CNTF: Ciliary neurotrophic factor
ERG: Electroretinography
ETDRS: Early treatment of diabetic retinopathy study
EZW: Ellipsoid zone width
FPDI: Fundus perimetry deviation index
GFs: Growth factors
NGF: Neural growth factor
OCTA: Optical coherence tomography angiography
ORT: Outer retinal thickness
RP: Retinitis pigmentosa
RPE: Retinal pigment epithelium
WJ-MSC: Wharton’s jelly-derived mesenchymal stem cell
VF: Visual field
